# Proportion of Ophthalmic Self-Medication and Associated Factors among Adult Ophthalmic Patients Attending Borumeda Hospital, Dessie, Northeast Ethiopia

**DOI:** 10.1155/2020/6932686

**Published:** 2020-04-25

**Authors:** Nebiyat Feleke Adimassu, Zemed Guchma Woldetsadik, Haile Woretaw Alemu

**Affiliations:** ^1^Department of Optometry, School of Medicine, College of Medicine and Health Science, University of Gondar, Gondar, Ethiopia; ^2^Ophthalmology Department, Boru Meda Hospital, Dessie, Amhara, National Regional State, Ethiopia

## Abstract

**Purpose:**

The aim of this study was to determine the proportion of ophthalmic self-medication and associated factors among adult ophthalmic patients attending Borumeda Hospital, Northeast Ethiopia, 2019.

**Methods:**

An institution-based cross-sectional study design was conducted on 402 participants at Borumeda Hospital from April 29 to May 24, 2019. Systematic random sampling technique was used to get study participants. Data were collected with a face-to-face interview by using a semistructured questionnaire. Data were entered into Epi Info 7 and analyzed by SPSS 20. Descriptive statistics and binary logistic regression analysis were employed. *P* values of less than 0.05 were considered statistically significant.

**Results:**

The proportion of ophthalmic self-medication was 28.6% (95% CI; 24.6–33.3). Age-group 29–42 years (AOR: 2.19, 95% CI: 1.06–4.52), absence of health insurance (AOR: 4.29; 2.35–7.84), more than 10 kilometer traveling distance to get eye services (AOR: 3.11; 1.58–6.12), previous experience of ocular illness (AOR: 2.62, 95% CI: 1.53–4.48), family or friend experience of ocular illness (AOR: 2.65, 95%CI: 1.43–4.92), availability of ophthalmic medicine bottle/tube at home (AOR: 4.59, 95% CI: 2.36–8.92), and poor knowledge about hazards of self-medication (AOR: 6.22; 3.26–11.85) were significantly associated with ophthalmic self-medication. *Conclusion and Recommendations*. The proportion of ophthalmic self-medication was high, which needs stakeholders' attention. The policymakers and regulatory body better to scale-up health insurance coverage, nearby accessible eye care services, improve knowledge of patients regarding the effect of ophthalmic self-medication, and proper disposal of leftover eye medication from the house. It is better to take regulatory actions on those who dispense ophthalmic medications without prescription.

## 1. Introduction

Self-medication is defined as selection and use of medicines by individuals to treat self-recognized illnesses or symptoms [1]. Ophthalmic self-medication is also defined as obtaining and consuming one or more medications without the advice of the eye care professional. This behavior includes purchasing drugs without a prescription, using leftover doses from previous prescriptions, and sharing drugs with other family members [2].

People practice ophthalmic self-medication for a variety of reasons. Ocular symptoms like redness, watering of the eye, foreign body sensation, and itching of the eye are common symptoms that initiate to use ophthalmic self-medication. In addition to this, previous ocular illness, living far from the hospital, unaware and poor knowledge about hazards of self-medication, and simplifying their illness are among the factors for ophthalmic self-medication [2–6].

Globally, the prevalence of ophthalmic self-medication ranges from 25.6% to 73.6% [2–9]. The prevalence of self-medication has sharply increased throughout the world. It is common practice in developing countries. Worldwide from all drugs purchased without any prescription, 80% accounted by developing countries [10]. In Ethiopia, eye diseases were common illnesses that lead to self-medication [11–15].

Self-medication practice is highly prone to irrational use of medications. The study showed that almost 90% of the ophthalmic self-medication was used inappropriately [2]. Possible consequences of improper administration of ophthalmic medication include microbial resistance, poor treatment outcome, adverse reactions, and disease progression, and finally may lead to visual morbidity [7, 16]. When medications such as steroids are misused, they aggravate existing infections and may lead to blindness [17]. In addition, self-medication practice negatively affects healthcare-seeking behavior of individuals and causes delay in diagnosis of problems and appropriate treatments [15].

Even though various studies were conducted on self-medication practices in different parts of Ethiopia, there was limited published evidence specifically on ophthalmic self-medication practice and factors that influence it among ophthalmic patients.

Therefore, this study aimed to determine the proportion of ophthalmic self-medication practice and associated factors among adult ophthalmic patients attending Borumeda Hospital, Northeast Ethiopia. The result of this study will be important as a baseline for the researcher and a source of information for policymakers as well as eye health care provider for appropriate interventions to prevent the risks associated with self-medication practice.

## 2. Methods

### 2.1. Study Design, Setting, and Sampling

An institution-based cross-sectional study was conducted to assess the proportion and associated factors of ophthalmic self-medication practice among adult ophthalmic patients attending Borumeda Hospital, Northeast Ethiopia, 2019.

The study was conducted at Borumeda Hospital from April 29 to May 24, 2019. Borumeda Hospital is found in Dessie town. Dessie town is located in 401 km far from Northeast of Addis Ababa, the capital city of Ethiopia. It has two public hospitals, four private hospitals, one private eye clinic, three government health centers, and thirty-six private clinics, and 56 drug retail outlets [18, 19].

Borumeda Hospital was established in 1955 G. C by Missionary to give ophthalmology and dermatology services. It has a catchment of approximately 2.5 million people according to the data obtained from the hospital office.

The secondary eye care unit has two ophthalmologists, five optometrists, one cataract surgeon, and two ophthalmic nurses. It provides comprehensive eye care services including major and minor ocular surgery, refraction service, and outpatient and inpatient service.

A total of 407 sample size was determined by a single proportional formula by considering 10% nonresponse rate. Then, a systematic random sampling method was used to select participants with a sampling fraction of three (every 3^rd^ individual).

The study was conducted in accordance with the Declaration of Helsinki and approved by the University of Gondar Ethical Review Board. In accordance with the Ethiopian National Research, Ethics Review Guideline, verbal informed consent was obtained from all adults older than 18 years using an information sheet in the local language “Amharic.” Since the study did not involve invasive eye examination procedures, the university ethical review board approved verbal informed consent. Permission was obtained from Borumeda Hospital, and verbal consent was obtained from each study participants after explaining the purpose of the study. The interview was taken after the treatment. Participants got proper treatment whether they refused or agreed for the interview. They have the full right to participate and to refuse or withdraw at any time they want. Confidentiality of the information obtained was assured by coding and locking the data. Confidentiality was maintained during the data collection and analysis procedure.

The study participants' agreements were first obtained verbally prior to data collection. Then, the data were collected by trained senior optometrists who came from other institutions away from the hospital where the study was conducted.

All participants were informed about the adverse effect of using ophthalmic medication without consultation of the eye care professional.

### 2.2. Definition of Ophthalmic Self-Medication

#### 2.2.1. Ophthalmic Self-Medication Practice

The participant who used one of the ophthalmic modern medicines at least once within the past two years for the specific ocular problem without consultation of eye care professionals [20].

#### 2.2.2. Health Insurance

Participants who had health insurance from the government and those who had free medical services said to be health insured.

#### 2.2.3. Distance to Get Eye Care Service

What is the traveling distance from residence to get eye care service? Participants who traveled more than ten kilometers to get eye care service were considered to be far, and the participants who travel ten kilometers or less were considered to be near [21].

#### 2.2.4. Knowledge

Knowledge was determined based on ten knowledge questions and was graded as for its level (good and poor). The participant who answered correctly was given a score of one, and those did not answer correctly or did not know was given a score of zero. The total score was out of ten. A participant said to be having good knowledge if he/she answers ≥50% (≥5/10) of the question. Otherwise, it was considered as poor knowledge [13].

#### 2.2.5. Housewife

A female who has husband, not employed by governmental and nongovernmental employers, who spent their time at home to keep house clean, cook foods, and take care of the child was considered as a housewife.

### 2.3. Data Collection

The pretested and structured questionnaire of local language “Amharic” was used to carry out an interview with adults older than 18 years (S1 questioner). Regular check-up for completeness and consistency of the data was made on a daily basis. On the fieldwork, data quality was ensured through 5% on spot cross-checking of the sample at hospital by principal investigators. The collected data have been checked for accuracy and completeness by the principal investigators. The questioner included questions to assess sociodemographic factors, socioeconomic factors, healthcare-related factors, knowledge, and practice of ophthalmic self-medication.

### 2.4. Statistical Analysis

After coding, the data were entered into Epi Info version 7, exported, and analyzed by using SPSS version 20. Both descriptive and analytical methods were employed for analysis. Summary statistics, frequencies, and cross-tabulations were performed for the descriptive analysis of the data. Bivariable and multivariable logistic regression was used to determine the associated factors. The variables that were found with *P* < 0.2 at bivariable logistic regression were entered to multivariable analysis. Variables were fitted into the model by using the enter method. The Hosmer and Lemeshow model fitness was used to check the model fitness of data. Multicollinearity between the independent variables was checked by the variance inflation factor. Adjusted odds ratio with a 95% confidence interval was used to show the strength of association. Those variables with a *P* value less than 0.05 were considered as statistically significant.

## 3. Results

### 3.1. Sociodemographic Characteristics of the Study Participants

A total of 402 adults participated with a response rate of 98.7%. The median age was 42 years with an interquartile range of 32 years. The majority (245, 60.9%) of the respondents were males. One hundred eight (26.9%) of the respondents were in the age-group 18–28 years. One hundred thirty-four (33.3%) of the population were unable to read and write (Table 1).

### 3.2. Health Service and Previous Ocular History-Related Characteristic of Study Participants

One hundred ninety-six (48.8%) of the study participants had health insurance. Majority 300 (74.6%) of the participants were traveled greater than ten km distance to get eye care services. One hundred sixty-five (41.0%) of the participants had the previous history of eye illness and about 79 (19.7%) of the participants had a positive history of family or friends ocular illness. Sixty-three (15.7%) of participants had an eye medication bottle/tube at their own home. All participants heard about the hazards of self-medication and 321 (79.9%) of participants had good knowledge about the hazards of self-medication.

### 3.3. Proportion of Ophthalmic Self-Medication Practice

The proportion of ophthalmic self-medication practice was 115 (28.6%) (95% CI: 24.6–33.3). Among them, 100 (87.0%) did not know the name of the medication they had used and 95 (82.6%) did not check the expiry date of the drug before they use it. Regarding the effects of the medication, the majority (81, 70.4%) of self-utilized participants did not improve from their illness, 20 (17.4%) of participants improved from their illness, and 14 (12.2%) of participants were worse their problem after self-medication.

### 3.4. Symptoms of Participants Triggered Ophthalmic Self-Medication

The common symptoms/illnesses that initiated for self-medication were redness, blurring of vision, and itching (Table 2).

### 3.5. Source of Ophthalmic Medicines for Self-Medication

The major sources of ophthalmic medicine for self-medication were pharmacy house (83 (72.2%)), followed by leftover medication (20 (17.4%)). The other source of medicine for ophthalmic self-medication was borrowing from friends or family 12 (10.4%).

### 3.6. Reasons for Self-Medication

**Figure 1:**
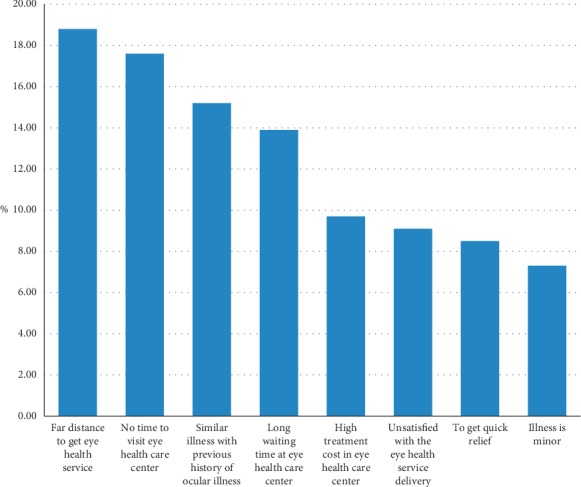
Reason of study participants for self-medication among adult ophthalmic patients attending Borumeda Hospital, Northeast Ethiopia, June 2019 (*n* = 165).

The major reason for ophthalmic self-medication was the far distance to get eye health care service (18.8%), followed by no time to visit at the eye health care center (17.6%) (Figure 1).

### 3.7. Factors Associated with Ophthalmic Self-Medication Practice

Binary logistic regression analysis showing age-group 29–42 years, absence of health insurance, previous history of ocular illness, family or friends experience of ocular illness, availability of eye medication bottle/tube at home, far traveling distance to get eye care services, and poor knowledge about self-medication were significant factors associated with ophthalmic self-medication practice.

Respondents of age-group 29–42 years were 2.19 times (AOR: 2.19, 95% CI: 1.06, 4.52) more likely to practice ophthalmic self-medication than those of age-group 18–28 years.

Ophthalmic patients who did not have health insurance were more than four times (AOR: 4.29, 95% CI: 2.35–7.84) more likely to practice self-medication than those who had health insurance. Ophthalmic patients who traveled more than ten km to get eye health services were nearly three (AOR: 3.11, 95% CI: 1.58–6.12) times more likely to practice self-medication than those who traveled less than or equal to ten km.

Ophthalmic patients with previous experience of ocular illness were more than two times (AOR: 2.62, 95% CI: 1.53–4.48) more likely to practice ophthalmic self-medication than those without previous experience of ocular illness. Adult ophthalmic patients with family or friend experience of ocular illness were nearly three (AOR: 2.65, 95% CI: 1.43–4.92) times more likely to practice self-medication than those without family or friend experience of ocular illness. Ophthalmic patients who had ophthalmic medication (bottle/tube) at home were more than four times (AOR: 4.59, 95% CI: 2.36–8.92) more likely to practice self-medication than those who had not available ophthalmic medicines at home.

Adult ophthalmic patients who had poor knowledge about the hazards of self-medication were more than six (AOR: 6.22, 95% CI: 3.26–11.85) times more likely to practice self-medication than those who had good knowledge (Table 3).

## 4. Discussion

In this study, the proportion of ophthalmic self-medication practice was 28.6% (95% CI: 24.6, 33.3). This finding is in line with a study from Argentina (25.6%) [20] and Colombia (25.7%) [22]. This might be due to the similarity of target population in terms of age. These studies were carried on the adult population. They also considered modern ophthalmic medication use to define self-medication.

This finding is lower than the report from Tanzania (59.8%) [5], Nigeria (73.6%) [2], India (41.2%) [23], and Brazil (40.5%) [7]. This variation might be due to the difference in the target population in terms of age and the use of traditional medicine included as self-medication practice. The previous study done in Tanzania [5], India [23], and Brazil [7] included both traditional and modern medicine use as self-medication, while in this study only modern medicine was considered. In addition to this, the target population of the study done in Nigeria [2] and Brazil [7] covered all age-groups including the pediatric population, while this study included only adult population (age > 18 years).

Regarding associated factors, respondents of age-group 29–42 years were more likely to practice self-medication than those of age-group 18–28 years. This might be due to eye diseases being more common in older people, and this could lead to ophthalmic medicines without consultation of eye-care providers [24].

Noninsured ophthalmic patients were more likely to practice self-medication than insured ophthalmic patients. This finding agrees with studies done in Ghana [6]. This might be due to the financial constraint that participants could not afford to pay all eye health services. In addition to this, having health insurance increase health care utilization, and this could reduce self-medication practice [25].

Ophthalmic patients who traveled a far distance to get eye health services were more likely to practice self-medication than those who traveled near distance. This might be due to inaccessibility of eye-care center around. In addition to this, it might be due to the financial constraint that participants might not afford transportation cost to come to the eye health center and may prefer to buy ophthalmic medicine from a nearby pharmacy.

Adult ophthalmic patients with a history of previous ocular illness were more likely to practice self-medication than patients without a previous history of ocular illness. This was supported by different studies [2, 6]. This might be due to the similarity of symptoms of eye diseases patients believing that ocular medications taken previously can cure their current eye disease.

Ophthalmic patients who had ophthalmic medication bottle/tube at home were more likely to practice self-medication than those who had not available ophthalmic medicines at home. This might be due to the similarity of symptoms of eye diseases patients believing that ocular medications taken previously can cure their current eye disease.

Those study participants with positive family/friend history of ocular illness were more likely to practice ophthalmic self-medication than those who had no family/friend history ocular illness. This might be due to the similarity of symptoms or illness leading to the use of similar ophthalmic medicine.

Adult ophthalmic patients who had poor knowledge about the hazards of self-medication were more likely to practice self-medication than those who had good knowledge. This finding was agreed with different studies [13, 22]. This might be due to knowledgeable ophthalmic patients fearing the bad adverse reactions of improper use of medication.

Since optometrists participated for data collection, this study has social desirability bias.

The study also has a limitation of recall bias because ophthalmic self-medication is assessed based on the self-report of the participants for their last two years of experiences. Income was not assessed by wealth index.

## 5. Conclusion and Recommendations

The proportion of ophthalmic self-medication was high, which needs stakeholders' attention. Absence of health insurance, history of previous ocular illness, family or friends experience of ocular illness, availability of eye medication bottle/tube at home, far-traveling distance to get eye care services, and poor knowledge about hazards of self-medication were significantly associated factors. The policymakers and regulatory body better to scale-up health insurance coverage, nearby accessible eye care services, improve knowledge of patients regarding the effect of ophthalmic self-medication, and proper disposal of leftover eye medication from the house. It is better to take regulatory actions on those who dispense ophthalmic medications without prescription.

## Figures and Tables

**Table 1:** Sociodemographic characteristics of the study participants attending Borumeda Hospital, Northeast Ethiopia, June 2019 (*n* = 402).

Characteristic	Frequency	Percent
*Sex*		
Male	245	60.9
Female	157	39.1

*Age* (years)		
18–28	108	26.9
29–42	101	25.1
43–60	100	24.9
≥61	93	23.1

*Religion*		
Christian	202	50.2
Muslim	200	49.8

*Residence*		
Urban	213	53.0
Rural	189	47.0

*Marital status*		
Currently single	112	27.9
Currently married	290	72.1

*Level of education*		
Unable to read and write	134	33.3
Read and write only	58	14.4
Primary education	73	18.2
Secondary education	53	13.2
College and above	84	20.9

*Type of occupation*		
Farmer	162	40.3
Government employee	50	12.4
Merchant	66	16.4
Housewife	39	9.7
Student	42	10.5
Daily laborer	31	7.7
Other^*∗*^	12	3.0

*Family monthly income* (ETB)		
≤720	103	25.6
721–965	98	24.4
966–3000	111	27.6
≥3001	90	22.4

^*∗*^Work seekers and drivers.

**Table 2:** Symptoms of study participants for ophthalmic self-medication practice among study participants attending Borumeda Hospital, Northeast Ethiopia, June 2019 (*n* = 119).

Symptoms	Frequency	Percent
Redness	47	39.5
Blurring of vision	29	24.4
Itching	26	21.8
Foreign body sensation	8	6.7
Others^*∗*^	9	7.6

^*∗*^Burning sensation, swelling, and discharge.

**Table 3:** Factors associated with ophthalmic self-medication among adult ophthalmic patients attending Borumeda Hospital, Northeast Ethiopia, June 2019 (*n* = 402).

Variables	Ophthalmic self-medication		COR (95% CI)	AOR (95% CI)
	Yes	No		
*Age-group* (years)				
18–28	25	83	1.00	1.00
29–42	35	66	1.76 (0.96, 3.22)	2.19 (1.06, 4.52)^*∗*^
43–60	34	66	1.71 (0.93, 3.14)	1.69 (0.80, 3.57)
≥61	21	72	0.96 (0.50, 1.87)	0.89 (0.38, 2.09)

*Health insurance*				
No	77	129	2.48 (1.57, 3.90)	4.29 (2.35, 7.84)^*∗∗∗*^
Yes	38	158	1.00	1.00

*Previous history of ocular illness*				
Yes	67	98	2.69 (1.72, 4.19)	2.62 (1.53, 4.48)^*∗∗*^
No	48	189	1.00	1.00

*Family or friends experience of ocular Illness*				
Yes	39	40	3.16 (1.90, 5.28)	2.65 (1.43, 4.92)^*∗∗*^
No	76	247	1.00	1.00

*Availability eye medication bottle/tube at home*				
Yes	39	24	5.62 (3.18, 9.93)	4.59 (2.36, 8.92)^*∗∗∗*^
No	76	263	1.00	1.00

*Travel distance to get eye care services*				
Far	94	206	1.76 (1.02, 3.01)	3.11 (1.58, 6.12)^*∗∗*^
Near	21	81	1.00	1.00

*Knowledge*				
Poor	44	37	4.18 (2.51, 6.97)	6.22 (3.26, 11.85)^*∗∗∗*^
Good	71	250	1.00	1.00

^*∗*^ *p* ≤ 0.05; ^*∗∗*^*p* ≤ 0.01; ^*∗∗∗*^*p* ≤ 0.001; 1.00 = reference; CI = confidence interval.

## Data Availability

All data required are available on the manuscript and will be supporting information.
